# Social Isolation and Depression in Community Dwelling Older Adults

**DOI:** 10.1093/geroni/igaf122.1628

**Published:** 2025-12-31

**Authors:** Loretta Anderson, Lenis Chen-Edinboro, Denise Orwig, Fernando Wagner

**Affiliations:** University of Maryland School of Medicine, Baltimore, Maryland, United States; The University of North Carolina at Wilmington, Wilmington, North Carolina, United States; University of Maryland, Baltimore, Baltimore, Maryland, United States; University of Maryland, Baltimore County, Baltimore, Maryland, United States

## Abstract

Studies show that 25% of older adults experience social isolation and approximately 28% experience depression, and both are linked to increased risk of chronic health conditions and poor health outcomes. This study examines the association between social isolation and depression in a nationally representative sample of community-dwelling older adults. Using the 2011 National Health and Aging Trends Study (NHATS), logistic regression models assessed the cross-sectional association between social isolation and depression, accounting for common sociodemographic factors and health conditions. Social isolation was measured using a previously validated measure in NHATS, and depression was measured using the PHQ-2. The analytic sample for this study included 6,802 participants aged 65 and older. Fully adjusted models showed mild social isolation having 24% greater odds of depression [OR = 1.24 (95%CI: 1.07,1.43), p < 0.01] compared to those without social isolation. For severe social isolation, bivariate analysis showed borderline significance of 37% greater odds of depression compared to those without social isolation [OR = 1.37 (95%CI: 0.99,1.89), p = 0.054], and became insignificant when controlling for sociodemographic and health factors. Mild social isolation was associated with depression in community-dwelling older adults, but severe social isolation was not. Longitudinal studies are warranted to confirm this difference between mild and severe social isolation and their association with depression in older adults. These results remain important as an increasing number of older adults in the United States experience social isolation. These findings may help inform clinical and policy efforts to improve depressive symptoms by helping to reduce social isolation in older adults.

